# OF spine classification of osteoporotic thoracolumbar vertebral body fractures by MRI and conventional radiographs only leads to high inter-observer agreement rates-an additional CT adds limited information for the of classification and the OF score

**DOI:** 10.1186/s12891-022-06056-4

**Published:** 2022-12-12

**Authors:** Ulrich J. Spiegl, Lars Behr, Georg Osterhoff, Gunnar Rupprecht, Max J. Scheyerer, Sebastian Katscher

**Affiliations:** 1 Department of Orthopaedics, Trauma and Plastic Surgery, University Hospital Leipzig, Liebigstr. 20, 04103 Leipzig, Germany; 2 Interdisciplinary Spine Center and Neurotraumatology, Sana Hospital Leipziger Land, Borna, Germany; 3 Department for Orthopaedic and Trauma Surgery, University of Düsseldorf, University Hospital Düsseldorf, Düsseldorf, Germany

**Keywords:** OF spine classification, OF spine score, MRI, CT, Osteoporotic vertebral body fractures

## Abstract

**Objectives:**

The purpose of this study was to investigate whether the addition of computed tomography (CT) to magnetic resonance imaging (MRI) improves the accuracy of classifying osteoporotic vertebral body fractures (OVBF).

**Methods:**

A retrospective analysis of a prospective single-center database has been performed. All consecutive patients who had suffered an acute thoracolumbar OVBF in one level II spine center between 2017 and 2019 were analyzed. Thereby, fractures of type OF 1 and OF 5 were excluded. All fractures were initially classified by 5 board-certified orthopaedic surgeons based on MRI and conventional radiographs. Afterwards a reclassification including CT scans were performed. Differences in OF classification and OF score values between both measurements were analyzed.

**Results:**

A total of 61 patients were analyzed with a mean age 75.8 years (SD: 8.8 years). In 82.9% of the cases, there was no difference in OF classification comparing classification based only on MRI versus MRI + CT. A difference of more than two OF types was observed in less than 1% of all ratings. The inter-rater reliabilities of the OF classification based on CT + MRI and MRI alone were 0.63 and 0.49, respectively. In 97.5% of the cases there was no therapy-relevant difference of the treatment recommendation with respect of a surgical or nonoperative treatment recommendation based on the OF score.

**Conclusion:**

In terms of the OF classification and the OF score, the addition of CT add limited value compared to conventional radiographs and MRI only. Additionally, there is only a minor rate of disagreement in treatment recommendations when adding a CT.

## Introduction

The prevalence of thoracolumbar osteoporotic vertebral body fractures (OVBF) is high, ranging between 18 to 26% in Europe with a noticeable increase over the past decades [1]. A reliable and comprehensive fracture classification can help to assign appropriate treatment strategies to certain injury patterns. Recently, the osteoporotic fracture (OF) classification of the spine has gained increasing acceptance among spine surgeons [2–4]. By including clinical parameters such as the level of mobilization and pain situation, the OF score can be generated based on the OF classification to support the decision making process [3]. Generally, the OF classification consists of five fracture types. In addition to conventional radiographs, computed tomography (CT) and magnetic resonance imaging (MRI) were recommended in the first description of the classification [2]. However, the combination of CT and MRI are time consuming and associated with high costs. That MRI is essential for the diagnosis of thoracolumbar OVBF has been shown clearly [5]. Additionally, CT imaging may help to visualize the fracture morphology in more detail and has been recommended for traumatic vertebral body fractures in patients with normal bone quality [6]. However, no study has analyzed the need of additional CT imaging to correctly diagnose and classify thoracolumbar OVBF. By being able to abstain from an additional CT examination, the use of resources could be minimized, and both costs and the radiation exposure could be reduced.

Thus, the purpose of this study was to investigate whether the addition of computed tomography (CT) to magnetic resonance imaging (MRI) improves the accuracy of classifying osteoporotic vertebral body fractures (OVBF) by the OF spine classification and the effect of this on the treatment recommendation based on the OF score. We hypothesized, that a CT examination is not always necessary for proper fracture classification in patients with suspicion of OVBF. We try to clarify in which cases CT examination is mandatory and in which cases conventional radiographs in combination with MRI are sufficient to classify OVBFs correctly.

## Methods

All patients who suffered from an acute thoracolumbar OVBF in a single spine center were consecutively and prospectively documented as part of a prospective multicenter study between October 2017 to December 2019. The study was approved by the institutional ethics committee. The authors declare that the study was performed according to the ethical principles of the Declaration of Helsinki. All patients received conventional radiographs on the day of admission and a CT scan on the same day or the day after admission. All patients without contraindications received a total spine MRI including short tau inversion recovery (STIR) sequences. Initially, non-operative treatment was started in all patients without highly unstable fractures, including mobilization under sufficient analgesia without an orthosis. Follow-up radiographs were initiated after 3 to 5 days. In accordance with the recommendations of Blattert et al. [3], all parameters which are part of the OF score (Table 1) were documented at the day of decision making after the follow-up radiographs. This includes the level of mobilization, the Visual Analogue Score of pain (0–10; 0: no pain, 10: worst pain), screening for osteoporosis by DEXA-scan or Hounsfield units [7, 8], any fracture-related neurologic deficit, ongoing fracture progress based on the bisegmental kyphotic angle and the following risk factors: ASA status > 3, dementia, body mass index (BMI) < 20 kg/m^2^, nursing case, anticoagulation [3]. The parameters were documented as part of the prospective multicenter study by one of the authors (GR).

**Table 1:** Definition of the OF-Score in accordance to Blattert et al. [3]

OF-Score		
**Parameter**	**Grade**	**Points**
Fracture classification type (OF 1 -5)	1 – 5	2—10
Bone mineral density	T-score < -3	1
Ongoing fracture process	Yes; No	1; -1
Pain (under analgesia)	VAS ≥ 4; < 4	1; -1
Neurological deficit	Yes	2
Mobilization (under analgesia)	No; Yes	1;—1
Health status	ASA > 3; dementia; BMI > 20 kg/m^2^; nursing case; anticoagulation	Each -1; Max. -2

0–5 points: nonsurgical; 6 points: nonsurgical or surgical; > 6 points: surgical

*Abbreviations*: *ASA* American Society of Anesthesiologists risk classification, *BMI* Body mass index, *VAS* Visual analogue scale for pain, *Max* Maximum

**Fig. 1:**
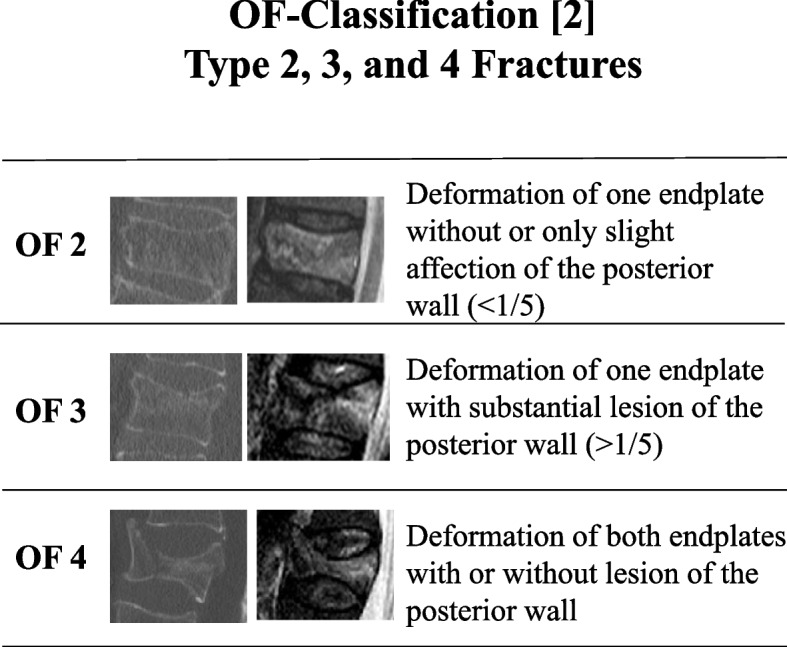
The Classification of OF types 2–4 are presented. OF type 2 fractures are defined by a deformation of one endplate with no or only minor involvement of the posterior wall (< 1/5). OF type 3 fractures affect one endplate only, but shows distinct involvement of the anterior and posterior wall (incomplete burst fractures). OF type 4 fractures affect both endplates with a loss of integrity of the vertebral frame structure, or vertebral body collapse, or pincer-type fracture. Thus, this fracture type includes complete burst fractures, split fractures (pincer type fractures), and vertebra plana

Patients without an MRI scan and all patients with type OF 1 and OF 5 fractures were excluded. Fractures of type OF 2–4 fractures are depicted in Fig. 1. In patients with more than one acute thoracolumbar OVBF, the fracture with the highest instability was selected for classification.

### Classification

All fractures were classified by a total of 5 raters. All raters were board-examined orthopaedic surgeons with experience in spine surgery. All classifications were performed independently by evaluation of the same diagnostic images. First, the OF classification was performed based in MRI and conventional radiologic images only. In a next step, classification was repeated based on conventional radiographs, MRI and the CT imaging of the fractured vertebral body.

The primary outcome parameter is the rate of agreement and disagreement in the OF classification classified by MRI alone versus MRI + CT between the raters. Secondary outcome parameters are the rate of agreement and disagreement of the OF score classified by MRI alone versus MRI + CT between the raters particularly with respect of treatment recommendation as well as the inter-rater reliabilities.

### Statistical analysis

Statistical analyses were performed using IBM SPSS Statistics for Windows, version 27.0 (IBM Corp., Armonk, N.Y., USA). Statistical analysis was made using descriptive statistics. First OF classifications using MRI and conventional radiographs (MRI) and additional CT (MRI + CT) of all raters were compared. Fractures with optimal agreement and those without agreement were analyzed separately. Next inter-rater reliability (IRR) was analyzed. Fleiss´ kappa (κF) was used to analyze IRR for the OF classification done by MRI and MRI + CT. In interpreting the κF values, the Landis and Koch criteria were used to indicate agreement (slight: 0.01–0.20, fair: 0.21–0.40, moderate: 0.41–0.60, substantial: 0.61–0.80, and almost perfect: 0.81–1.00) [9]. Finally, all treatment recommendations based on the OF score of all raters were compared between those that have been done using MRI or MRI + CT.

## Results

A total of 61 patients were included. The average age was 75.8 years (SD: 8.8 years, range 52 to 91 years), 44 (72.1%) were female. Based on MRI and CT, the senior author (SK) classified all fractures including 8 OF type II fractures (13.1%), 40 OF type III fractures (65.6%), and 13 OF type IV fractures (21.3%). This was defined as the gold standard. The mean pain level on the day of decision making was 6.2 (SD 2.1; range: 0 – 10).

**Fig. 2:**
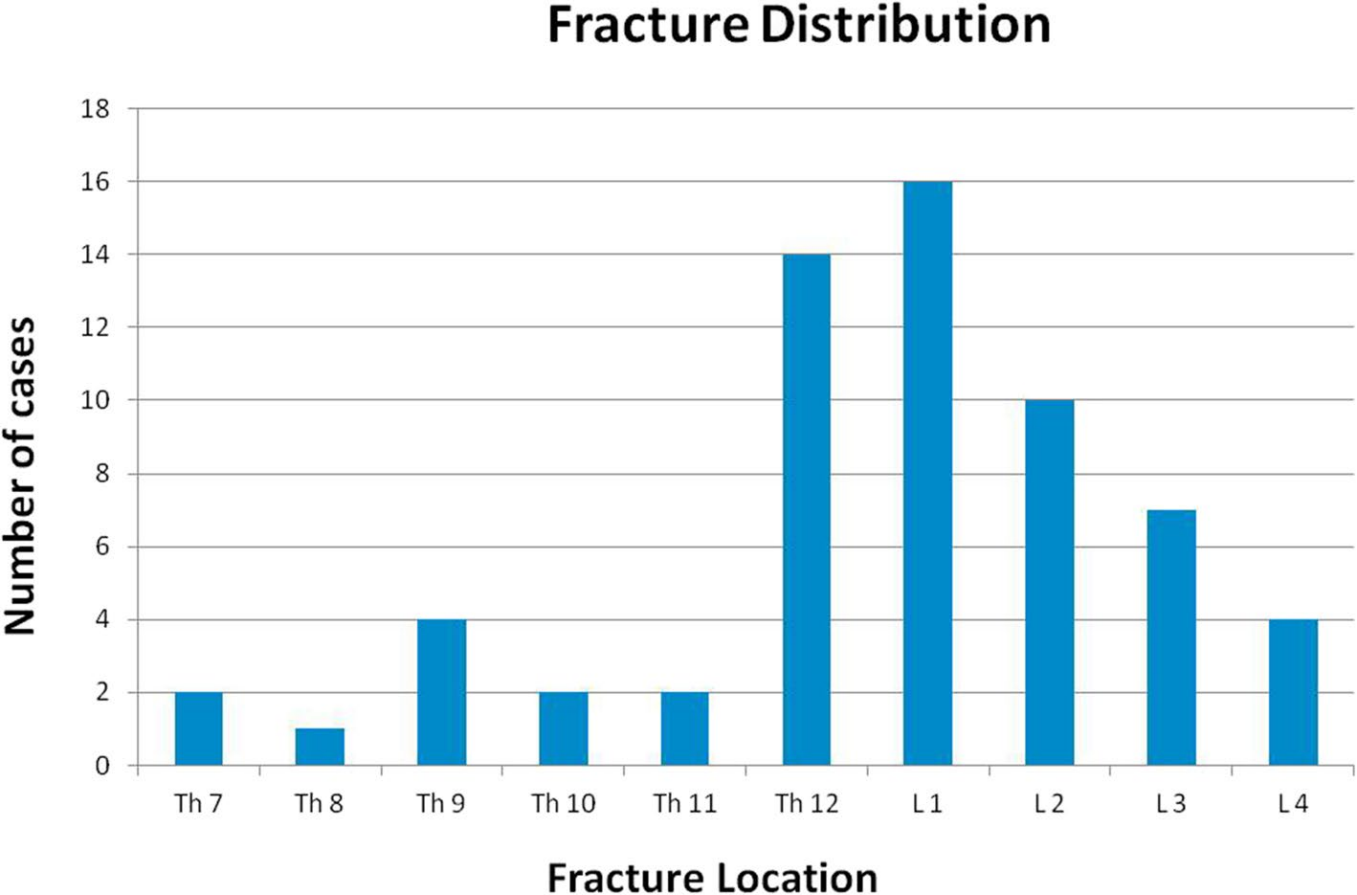
The fracture locations of all patients are depicted in this diagram. Fractured occurred between the 7th thoracic and 4th lumbar vertebral body with a majority at the thoracolumbar junction

The fracture locations are depicted in Fig. 2.

**Fig. 3:**
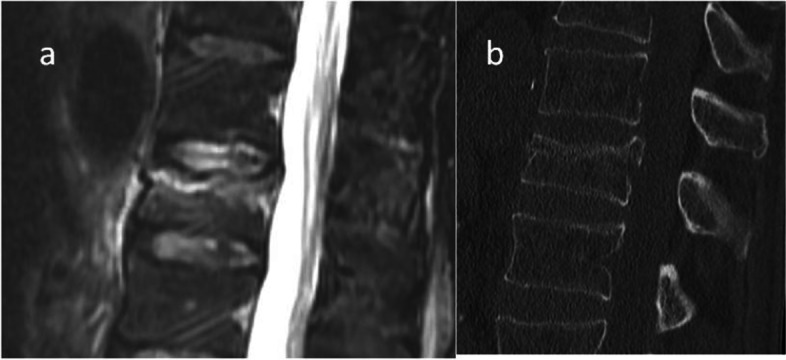
Depicted is a case with an incomplete burst fracture type OF 3. There was a perfect agreement for both MRI only (**a**) and in combination with MRI and CT (**b**)

**Fig. 4:**
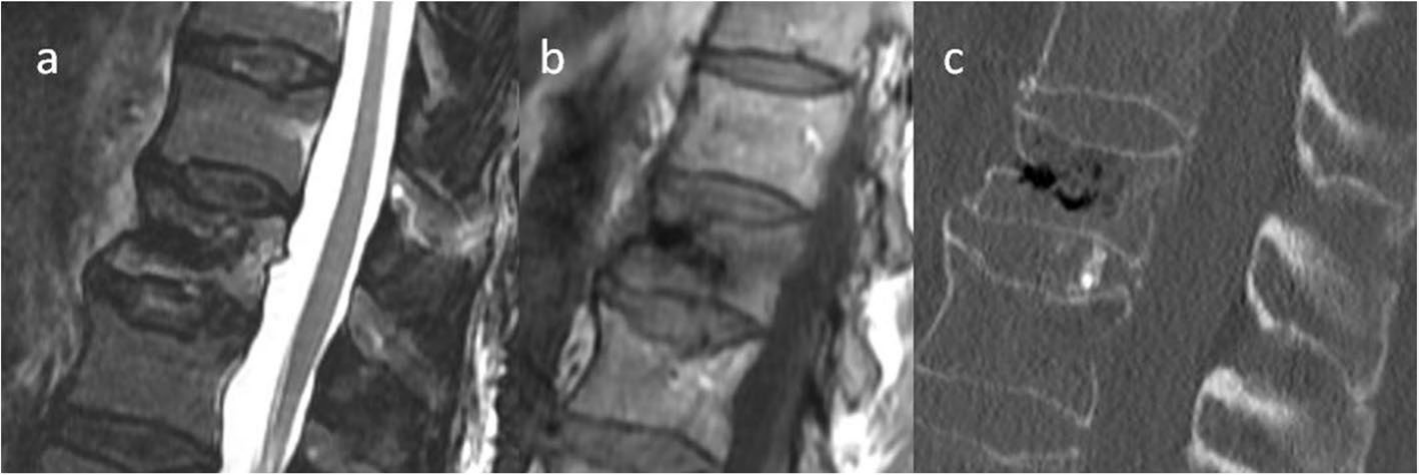
Depicted is a case with high disagreement between the classification done by MRI (**a**: STIR-Sequence, **b**: T2-Sequence) only and under consideration of both MRI and CT (**c**). The majority of rater classified this fracture as type OF 4 fracture after evaluating the MRI and re-classified it as type OF 3 fracture under consideration of the additional CT. However, the OF score was 7 and 9 in case of OF type 3 and OF type 4 fracture, respectively. Thus, operative treatment was recommended based on the OF-score regardless of type OF 3 or 4 fracture classification

The differences in the OF types classified by MRI alone versus MRI + CT between the raters are shown in Table 2. In 82.9% of the cases no difference could be seen. Differences of more than 2 OF types were found in less than 1% of all ratings. Figures 3 and 4 illustrate cases that showed total agreement and those with a high grade of disagreement.

**Table 2:** Difference between OF classification based on CT and MRI versus MRI alone (OF MRI)

	Rater I (%)	Rater II (%)	Rater III (%)	Rater IV^a^ (%)	Rater V (%)	Mean (%)
No Difference	90.2	82.0	75.0	78.7	88.5	82.9
OF MRI: -1	3.3	9.8	12.5	6.6	6.6	7.8
OF MRI: -2	0	1.6	0	0	1.6	0.6
OF MRI: + 1	6.6	6.6	12.5	13.1	3.3	8.4
OF MRI: + 2	0	0	0	1.6	0	0.3

*CT* Computer tomography, *MRI* Magnetic resonance imaging, *OF MRI* -1 defines an OF type classified by MRI alone that considered the fracture morphology less severe by one grade compared to the OF type classified by CT and MRI (MRI + CT) (example: OF type II classified by MRI versus OF type III classified by MRI + CT), *OF MRI* -2: MRI alone considered the fracture morphology less severe by two grades compared to the OF type classified by CT and MRI, *OF MRI* + 1: MRI alone considered the fracture morphology more severe by one grade compared to the OF type classified by CT and MRI, *OF MRI* + 2: MRI alone considered the fracture morphology more severe by two grades compared to the OF type classified by CT and MRI

^a^ General orthopaedic surgeon, no spine specialist

The inter-rater reliabilities of the OF classification with CT + MRI and MRI alone are shown in Table 3.

**Table 3:** The reliability of the classification based on CT and MRI as well as MRI alone

	MRT + CT		MRT		
		0.95 CI		0.95 CI	
	Kappa	(lower–upper limit)	Kappa	(lower–upper limit)	
**OF Spine (OF 2–4)**	0.630	0.549–0.711	0.494	0.416–0.572	
**OF 2**	0,342	0.237–0.446	0.209	0.106–0.311	
**OF 3**	0.614	0.510–0.718	0.487	0.385–0.590	
**OF 4**	0.802	0.698–0.906	0.676	0.573–0.778	

Fleiss’ Kappa for interrater reliability in evaluating the OF classification using MRI and CT (MRI + CT) versus MRI alone (MRI)

The differences in treatment recommendations with respect to surgical or nonoperative treatment based on the OF score including either the classification by MRI alone or MRI + CT are shown in Table 4. Generatlly, there was a high agreement for the diagnosis of OF 4 fractures using both MRI alone and MRI + CT (Table 3). In contrast, it seems to be difficult to differentiate between OF 2 and 3 fractures caused inferior agreement rates particularly when the classification was done by MRI only.

**Table 4:** Comparison of the treatment recommendation based on the OF score between MRI and MRI + CT

Treatment Recommendation	Rater I (%)	Rater II (%)	Rater III (%)	Rater IV (%)	Rater V (%)	Mean (%)
No Difference	88.5	82.0	82.0	82.0	88.5	84.7
No Relevant Difference ^X^	11.5	11.5	11.5	18.0	9.8	12.3
Relevant Difference	0	6.6	6.6	0	1.6	2.5

## Discussion

The main finding of this study was the high agreement between the OF classification based on MRI compared to MRI + CT, particularly under consideration of the treatment recommendation using the OF score. Thus, the classification based on MRI and radiographs only seems to be sufficient for the purpose of generating a correct treatment recommendation. Notwithstanding, the inter-rater RR was higher using MRI + CT compared to MRI alone.

These results appear to be controversial and need to be discussed in more detail.

Fractures of types OF 1 and OF 5 were excluded for this analysis, as OF 1 fractures are only visible in the MRI by definition [2] and as the concomitant tension band injuries OF 5 fractures are ideally visualized by MRI [10]. Thus, no beneficial effect of the CT for the identification of both OF 1 and OF 5 type fractures can be expected. CT might be particularly valuable to differentiate between OF type 2, 3, and 4 fractures [11, 12]. Interestingly, the agreement was still high and particularly for diagnosis of type 4 fractures with a substantial or almost perfect agreement for both MRI and MRI + CT, respectively. In contrast, the inter-rater RR of OF 2 fractures was only fair for both, MRI and MRI + CT. Notwithstanding, the agreement of the classification using MRI + CT was superior for all subtypes. These differences appear to be particularly relevant in the following two situations: First of all, fractures with only slight traumatic defect of the posterior wall in which a differentiation between a type 2 or type 3 fracture is difficult. Secondly, fractures with potential mild affection of the second endplate in which a differentiation between type 3 or type 4 fracture is hard. This can be particularly difficult in cases with central defects and a coronal split component without posterior wall affection. In these, the differentiation between type 2 und type 4 fractures can be difficult. This was visible in 3 of the patients in our collective. Based on the superior visualization of the CT to define fracture extent a superior inter-rater agreement was visible in these cases. However, these differences seem not to play a major role in the general treatment recommendation based of the OF score. On the one side, OF 2 or OF 3 fractures with little comminution are typically a domain of non-operative treatment without associated high instabilities and generally rather mild clinical courses. In contrast, severe OF 3 fractures with substantial lesions of the posterior cortex as well as type 4 fractures are commonly candidates for an operative treatment based on extended rates of fracture progress and the associated longer pain duration and limited mobilization. In contrast, fractures without severe posterior wall affection (type OF 2 or 3), but central vertebral body defects and potential affection of the second endplate (OF 4) might affect the treatment recommendation.

Thus, conventional radiographs with MRI alone seem to be sufficient for generating a treatment recommendation. However, an additional CT is useful in fractures in which the differentiation between OF 2 or 3 as well as between OF 3 and 4 cannot be done accurately particularly in those patients in which this difference would lead to a change in the treatment recommendation based on the OF score. Patients with OVBFs of type OF 2 or OF 3 and only minor pain and minor limitations as well as those with OVBFs of type OF 3 and OF 4 with major pain and limitations despite accurate treatment do not benefit from and additional CT examination with respect of the decision making. Additionally, CT evaluation is useful in those patients in which a differentiation between OF 2 and OF 4 fractures cannot be sufficiently performed. Hereby, an effect for the treatement recommendation based on the OF score is highly likely. Additionally, a CT is important if surgical treatment is indicated and preoperative planning cannot be done sufficiently or the treatment strategy may be unclear based on the conventional radiographs and the MRI findings.

Generally, the inter-rater reliability for MRI + CT (0.63) was identical to the inter-rater reliability reported by Schnake et al. [2]. It can be expected, that the inter-rater reliabilities might be even higher if type OF 1 and/or type OF 5 fractures were included based on the clear definition that can be identified by MRI. This needs to be proven by future studies.

Altogether, this study offers several limitations. First, the exclusion of OF 1 and 5 fractures is a limitation. However, the authors believe that this rather strengthens our conclusions based on the above mentioned reasons. Next, no second round of ratings of the same raters to evaluate the intra-rater reliability was performed. However, the almost identical inter-rater reliability reported by Schnake et al. [2] seems to confirm the external validity of this classification. Unfortunately, so far no global validation of the OF classification has been published.

In contrast, the rather high number of patients that were prospectively collected including the collections of all parameters that are necessary to calculate the OF score are the strengths of this study.

## Conclusion

In terms of the OF classification and the OF score, the addition of CT add limited value compared to conventional radiographs and MRI only. Additionally, there is only a minor rate of disagreement of in the treatment recommendation in accordance to the OF score between MRI and MRI + CT.

Thus, MRI in combination with conventional radiographs seem to be sufficient to gain an accurate treatment recommendation. However, an additional CT helps to increase the reliability of the classification and seems to be particularly helpful in cases where a differentiation between type 2 and 3 as well as type 3 and 4 fractures is difficult. If surgery is indicated, additional CT might be very useful for the purpose of preoperative planning.

## Data Availability

The datasets used and analyzed with this study are available on reasonable request from the corresponding author.
